# Prevalence of self-reported depression in Brazil: National Health Survey 2019 and 2013

**DOI:** 10.1590/SS2237-9622202200006.especial

**Published:** 2022-07-08

**Authors:** Valéria Cristina de Albuquerque Brito, Rafael Bello-Corassa, Sheila Rizzato Stopa, Luciana Monteiro Vasconcelos Sardinha, Catarina Magalhães Dahl, Maria Carmen Viana

**Affiliations:** 1 Ministério da Saúde, Departamento de Análise em Saúde e Vigilância de Doenças Não Transmissíveis, Brasília, DF, Brazil; 2 Organização Pan-Americana da Saúde, Brasília, DF, Brazil; 3 Universidade Federal do Espírito Santo, Programa de Pós-Graduação em Saúde Coletiva, Vitória, ES, Brazil

**Keywords:** Depression, Mental Disorders, Health Surveys, Cross-Sectional Studies

## Abstract

**Objective:**

To describe the prevalence of self-reported depression among Brazilian adults in the 2019 National Health Survey (PNS) and compare to the 2013 PNS.

**Methods:**

Cross-sectional study of Brazilian adults using data from the 2019 and 2013 PNS. Prevalence and 95% confidence intervals (95%CI) of self-reported depression were estimated by region and demographic characteristics. Bivariate analyses were conducted using chi-squared tests.

**Results:**

There were 90,846 participants aged ≥ 18 years in 2019, and 60,202 in 2013. Between 2013 and 2019, prevalence of self-reported depression increased from 7.6% (95%CI 7.2;8.1) to 10.2% (95%CI 9.9;10.6) and of individuals who sought healthcare, from 46.4% (95%CI 43.8;49.1) to 52.8% (95%CI 50.7;55.0). Private clinics were the main source of healthcare.

**Conclusion:**

Depression is highly prevalent in Brazil. Prevalence of diagnosis of depression and use of health services increased in the studied period. The predominance of care in private clinics suggests inequalities in the improvement of mental healthcare coverage.

**Study contributions**

**Main results**

One out of ten individuals reported depression diagnosis. There has been an increase in the prevalence of self-reported depression and in the pursuit of medical treatment for depression. Private services were the main source of care for depression.

**Implications for services**

The high proportion of people who sought care for depression in private establishments shows inequalities in access to services and requires the strengthening of the psychosocial care net-work to ensure equitable access to mental health care in Brazil.

**Perspectives**

The increase in the prevalence of depression diagnosis and in the pursuit of treatment among young adults, considering the limitations of self-reported diagnosis, endorses the necessity of specific studies focusing on the Brazilian population’s mental health.

## Introduction

Acknowledging the importance of mental and behavior disorders among the causes of morbidity and mortality in the global population prompted the inclusion of topics related to mental health and quality of life among the Sustainable Development Goals (SDG), endorsed by the United Nationes (UN).^1^ Estimates of the 2019 study named Global Burden of Disease (GBD) point out that, worldwide, 1 billion people suffered from mental disorders and substance abuse and addiction, representing 6% of the global burden of disease and more than 17% of the years lived with disability.^2^ It is also estimated that over 75% of persons that have mental disorders, including people with neurological conditions and those with disorders related to substance use, do not have access to mental health services, especially in low-income and middle-income countries.^3^

Depression is one of the conditions that contributes the most to the global burden of diseases related to mental health. In addition to being one of the main causes of disabilities worldwide, depression is associated with premature deaths due to suicide and other chronic diseases. Data from the 2019 GBD study estimated that over 270 million people suffered from depressive disorders, which, at that time, corresponded to approximately 3.8% of the global population.^2^ In Brazil, the prevalence of depressive disorders is estimated at 4.3%.^2^ The Brazilian component of the World mental health survey, the São Paulo Megacity mental health survey, conducted between 2005 and 2007, identified that the prevalence of depression throughout life and along the 12 months prior to the interview for all studies was 16.9% and 9.4%, respectively, among adults living in the Metropolitan Region of São Paulo.^4^ ^,^ ^5^

The investigation of the prevalence of depressive disorders and depressive symptoms in different population strata has been conducted by means of various methods, revealing the multi-faceted nature of research on mental health, a complex and stigmatized theme.^6^ In this context, research that identifies the diagnosis of psychiatric disorders, through directed self-report, enables evidencing not only the identification of symptoms by individuals, but also the pursuit of services and the recognition of the diagnosis in the health care network.^7^ Population surveys on the prevalence of self-reported depression represent an important resource with regard to conducting the surveillance of events related to mental health and associated factors, and can contribute to keeping track of access to diagnosis and treatment, enabling the development of more effective strategies for prevention and attention to people with mental disorders.

The inclusion of the investigation of self-reported depression in the National Health Survey (*Pesquisa Nacional de Saúde* - PNS) in 2013,^7^ and its maintenance in the 2019 edition, represent an important effort in monitoring the evolution of this health problem among the Brazilian population as a whole. In this sense, the objective of this study was to describe the prevalence of self-reported depression in the adult Brazilian population in the 2019 PNS, according to sociodemographic variables, and to compare the findings with the results of the 2013 PNS.

## Methods

This was a cross-sectional study on the prevalence of self-reported medical diagnosis of depression in Brazil, using data from the 2019 and 2013 PNS editions. The PNS is a national household survey, representative of the adult Brazilian population, conducted by the Brazilian Institute of Geography and Statistics (*Instituto Brasileiro de Geografia e Estatística* - IBGE), in partnership with the Ministry of Health. Data used in this study are in the public domain, available from the IBGE website, and were extracted on April 10, 2021. In 2013, the sample covered adults aged 18 years and older, while in 2019, the target population was comprised of individuals aged 15 years and older, residing in private households in Brazil.^8^

The survey used complex sampling design and participants were selected by means of multistage cluster sampling, in three stages: stratification of the primary sampling units (PSU), composed by one or more census tracts and random selection with a probability proportional to size (number of permanent private households in the census tracts); selection of the households in each PSU based on the most recent data available at the National Address File for Statistical Purposes (*Cadastro Nacional de Endereços para Fins Estatísticos* - CNEFE), through simple random sampling; followed by simple random sampling of one resident aged 15 years or over, based on the list of residents prepared at the time of the interview, with no substitution.

For the 2019 PNS, the sample size was calculated based on the selected indicators from the 2013 edition. From a total of 15,096 PSUs, 108,525 households were selected, with an expected number of 86,820 interviews.^8^ More in depth details on the sampling plan, data collection and weighting process were published by Stopa et al.^8^ and IBGE.^9^ For the 2013 PNS, the sample size was scaled based on expected estimates of indicators and desired coefficients of variation. From a total of 6,069 PSUs, 81,357 households were selected, considering an estimated loss of 23%. Details on the sampling plan and weighting of the 2013 PNS were published by Damacena et al.^10^ and Souza-Júnior et al.^11^

For this study, considering the differences in age groups between the 2019 and 2013 PNS samples, only respondents aged 18 years or older were included, in order to ensure the comparability of estimates.

Data related to self-reported depression were analyzed by means of the following question: *Has a doctor or mental health professional (such as a psychiatrist or psychologist) ever given you the diagnosis of depression?* (yes; no). Data pertaining to the last time the respondent received medical care for depression and the place of care were also used, through the questions: i) *When was the last time you received medical care due to depression?* (less than 6 months ago; 6 months to less than 1 year ago; 1 year to less than 2 years ago; 2 years and less than 3 years ago; 3 years ago or more; never received); and ii) *When you last received medical care for depression, where did that take place?* [pharmacy; basic healthcare unit (public health post or center or family health unit); public polyclinic, PAM (*Posto de Assistência Médica* - Medical Assistance center) or public Specialist Outpatient Clinic; UPA (*Unidade de Pronto Atendimento* - Emergency Care Unit), another type of public emergency care center (24/7), or emergency room or emergency department of a public hospital; public hospital outpatient clinic; private doctor’s office, private clinic or private hospital outpatient clinic; emergency room or emergency department of a private hospital; at home; other].

First, the prevalence and 95% confidence intervals (95%CI) were estimated for 2019, according to sociodemographic characteristics: sex (male; female); age group (18 to 29; 30 to 59; and 60 years old and over); schooling (no education and incomplete elementary school; complete elementary school and incomplete secondary school; complete secondary school and incomplete higher education; complete higher education); self-reported race/skin color [White and Black (Black and Brown)] and area of residence (urban; rural).

Bivariate analyses of the association between self-reported depression and the demographic characteristics of individuals were processed using the chi-square test, considering a significance level of 5%. Crude prevalence ratios of depression in relation to demographic variables and 95%CI were estimated using Poisson regression in order to assess differences in probability between strata of the demographic variables. Prevalence data among individuals with self-reported Indigenous or Yellow race/skin color were not presented, due to the lack of representativeness of the research for these groups.

Lastly, the prevalence of self-reported depression, of medical care for depression in the 12 months preceding the interview, and the proportional distribution of health services used for this type of care were descriptively compared by evaluating the estimates and their confidence intervals for both 2013 and 2019.

All the analyses were conducted using the survey module of the Stata software, version 14.2 (StataCorp. 2015. Stata Statistical Software: Release 14. College Station, TX: StataCorp LP), which takes into account weighting for complex samples.

Data related to both editions of the PNS are in the public domain and were approved by the National Research Ethics Committee, of the National Health Council, No. 328,159 for the 2013 edition, and No. 3,529,376 for the 2019 edition.

## Results

A total of 90,846 individuals aged ≥ 18 years participated in the 2019 PNS, and 60,202 in the 2013 PNS. The overall prevalence of self-reported depression in adults residing in Brazil, in 2019, was 10.2% (95%CI 9.9;10.6). Among the Brazilian regions, the South region had the highest prevalence (15.2%; 95%CI 14.2;16.2) and, among the states, the highest prevalence was observed in Rio Grande do Sul (17.9%; 95%CI 16.2;19.6). The lowest prevalences were observed in the North region (5.0%; 95%CI 4.4;5.6) and in the state of Pará (4.1%; 95%CI 3.0;4.1), as shown in Table 1.

**Table 1:** Prevalence of self-reported depression in the adult Brazilian population (≥ 18 years; n = 88,531), according to macro-region and Federative Units, National Health Survey, Brazil, 2019

Macro-region/Federative Units	n^a^	%	95%CI^b^
**North**	16,937	5.0	4.4;5.6
Rondônia	2,108	9.0	7.3;10.7
Acre	2,283	6.0	4.8;7.2
Amazonas	3,370	4.2	3.2;5.2
Roraima	2,135	5.1	3.9;6.3
Pará	3,696	4.1	3.0;5.2
Amapá	1,473	4.5	2.4;6.7
Tocantins	1,872	6.6	5.3;8.0
**Northeast**	30,702	6.9	6.5;7.3
Maranhão	4,889	5.4	4.7;6.2
Piauí	2,674	6.9	5.6;8.2
Ceará	4,141	8.1	7;9.2
Rio Grande do Norte	2,877	8.5	7.2;9.8
Paraíba	3,068	7.6	6.3;8.8
Pernambuco	3,992	6.8	5.7;7.8
Alagoas	2,898	6.2	5;7.4
Sergipe	2,563	8.5	7.4;9.7
Bahia	3,600	6.3	5.3;7.3
**Southeast**	19,435	11.5	10.8;12.2
Minas Gerais	5,128	13.7	12.1;15.2
Espírito Santo	3,463	11.3	9.8;12.8
Rio de Janeiro	4,849	8.1	7.1;9.0
São Paulo	5,995	11.8	10.6;12.9
**South**	11,276	15.2	14.2;16.2
Paraná	3,893	13.9	12.2;15.6
Santa Catarina	3,676	13.1	11.8;14.5
Rio Grande do Sul	3,707	17.9	16.2;19.6
**Midwest**	10,181	10.4	9.5;11.3
Mato Grosso do Sul	2,805	10.1	8.8;11.3
Mato Grosso	2,423	8.2	6.8;9.5
Goiás	2,648	12.0	10.3;13.8
**Distrito Federal**	2,305	9.4	7.8;11.0
**Brazil**	88,531	10.2	9.9;10.6

a) Unweighted values; b) 95%CI: 95% confidence intervals.

Higher rates were observed for the year 2019 among females (14.7%; 95%CI 14.1;15.4; p-value < 0.001), White race/skin color (12.5%; 95CI % 11.8;13.1; p-value < 0.001) and residents of urban areas (10.7%; 95%CI 10.2;11.1; p-value < 0.001). Regarding education, the highest prevalence was found among individuals with no schooling or with incomplete primary education (10.9%; 95%CI 10.3;11.5) and with complete higher education (12.2%; 95%CI 11.3;13.2). In terms of age groups, young adults (18 to 29 years old) had the lowest prevalence of depression (5.9%; 95%CI 5.2;6.7; p-value < 0.001), as shown in Table 2.

**Table 2:** Prevalence of self-reported depression in the adult Brazilian population (≥ 18 years; n = 88,531), according to sociodemographic characteristics, National Health Survey, Brazil, 2019

Variables	n^a^	%	95%CI^b^	PR^c^	95%CI^b^	p-value
**Sex**						
Male	1,930	5.1	4.7;5.5	1.0		< 0.001
Female	6,312	14.7	14.1;15.4	2.9	2.6;3.1	
**Age group (years)**						
18 to 29	836	5.9	5.2;6.7	1.0		< 0.001
30 to 59	5,040	11.3	10.8;11.9	1.9	1.7;2.2	
60 and older	2,366	11.8	11.1;12.6	2.0	1.7;2.3	
**Education**						
Complete higher education	1,614	12.2	11.3;13.2	1.0		< 0.001
Complete secondary education or incomplete higher education	2,363	9.0	8.4;9.6	0.7	0.7;0.8	
Complete primary education or incomplete secondary education	996	9.4	8.4;10.4	0.8	0.7;0.9	
No schooling or incomplete primary education	3,269	10.9	10.3;11.5	0.9	0.8;1.0	
**Race/skin color**						
Black (Black and Brown)	4,339	8.6	8.1;9.0	1.0		< 0.001
White	3,796	12.5	11.8;13.1	1.5	1.4;1.6	
**Place of residence**						
Rural	1,373	7.6	7.0;8.3	1.0		< 0.001
Urban	6,869	10.7	10.2;11.1	1.4	1.3;1.5	

a) Unweighted values; b) 95%CI: 95% confidence intervals; c) PR: Prevalence ratio.

**Figure 1:**
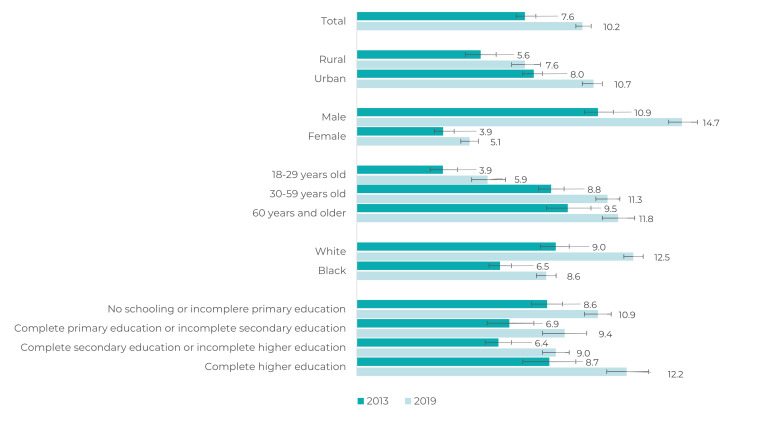
Prevalence (%)^a^ and 95% confidence interval of adults (≥ 18 years of age) with self-reported depression, according to sociodemographic characteristics, Brazil, National Health Survey, 2013 and 2019

There was an increase in the prevalence of self-reported depression in all categories of sociodemographic variables analyzed, between 2013 and 2019, from 7.6% (95%CI 7.2;8.1) to 10.2% (95%CI 9.9;10.6), respectively. This increase was more accentuated among females, people of White race/skin color, people with higher education and urban residents (Figure 1).

a) Weighted data.

**Figure 2:**
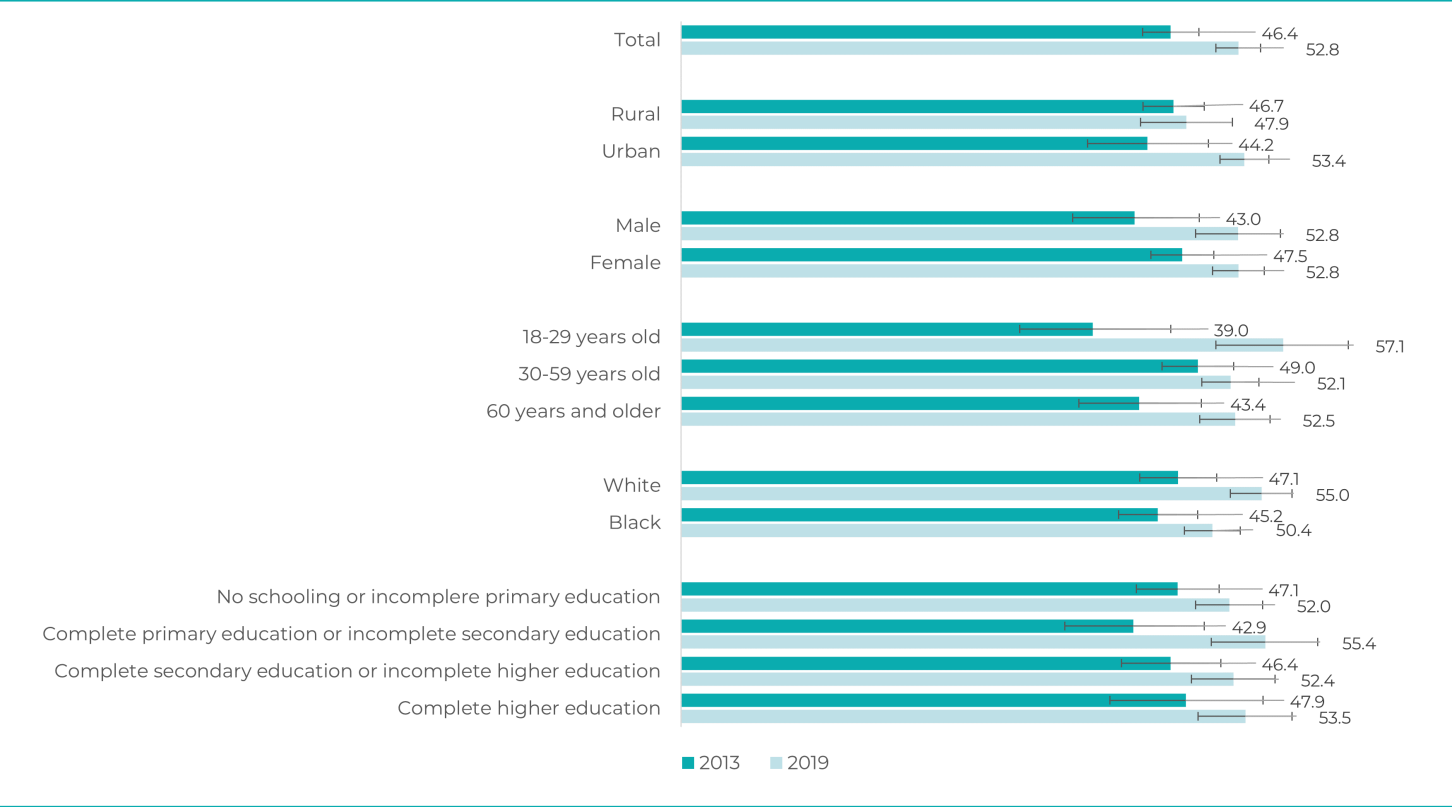
Prevalence (%)^a^ and 95% confidence interval of adults (≥ 18 years of age) with self-reported depression, who received medical care to depression (in the 12 months prior to the interview), according to sociodemographic characteristics, Brazil, National Health Survey, 2013 and 2019

a) Weighted data.

**Figure 3:**
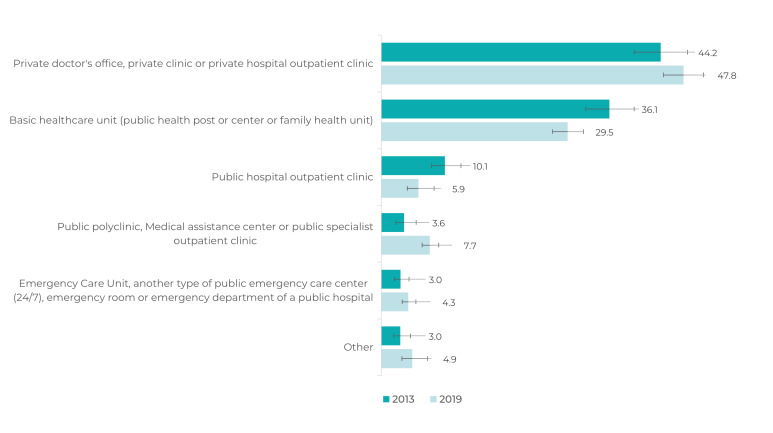
Proportional distributions (%)^a^ of sources of medical care due to depression (in the 12 months prior to the interview) among adults (≥ 18 years old) with self-reported depression, Brazil, National Health Survey, 2013 and 2019

There was also a significant increase in the prevalence of individuals who received medical care in the 12 months preceding the interview, from 46.4% (95%CI 43.75;49.1) in 2013 to 52.8% (95%CI 50.7;55.0) in 2019, especially among young adults (18 to 29 years old) and urban residents (Figure 2). Among these individuals, the majority (47.5%; 95%CI 44.7;50.2) sought medical care in private offices or clinics or outpatient clinics of private hospitals. Between 2013 and 2019, there was also a reduction in the proportions of individuals who received medical care in basic health care units and in public hospital outpatient clinics (Figure 3).

a) Weighted data.

## Discussion

Analyses of the data from the 2013 and 2019 PNS showed higher prevalence of self-reported depression in states in the South and Southeast regions, and lower in the North and Northeast regions, as well as a general increase in rates when comparing both surveys. Female individuals, of White race/skin color, with complete higher education and residing in urban areas stand out. Private doctor’s offices, private clinics and outpatient clinics of private hospitals corresponded to the main source of care among individuals who sought medical care in the 12 months prior to the interview; on the other hand, basic health care units were the second most frequent location, although with a reduction in the proportion of care provided in these locations in 2019.

Considering that this study evaluated the prevalence of self-reported medical diagnosis of depression, and not the prevalence of previous or clinically manifested depressive disorders at the time of the research, these differences in the distribution of prevalence between regions do not necessarily describe the distribution of individuals who have depressive symptoms. The data do represent the organization, availability and access of residents in different regions of Brazil to health professionals and mental health services, as well as the self-perception of health-disease and pursuit of treatment, factors that impact the potential for diagnosis.

The high prevalence observed in the South region is compatible with other studies,^12^ possibly correlated with the high rates of suicide and suicide attempts in that region,^13^ especially in Rio Grande do Sul.^14^ In addition, it may be related to regional differences in the planning of services and training programs for professionals to meet the demands in mental healthcare.

Fernandes et al.^15^ evaluated the coverage of Psychosocial Care Centers (*Centros de Atenção Psicossocial* - CAPS) in Brazil, demonstrating greater coverage in the South and Northeast regions, where these services cover, respectively, 57% and 55% of the population; while the North region had the lowest coverage, reaching only 35% of the population. It should be noted that CAPS offer specialized services and that the implementation of the Psychosocial Care Network (*Rede de Atenção Psicossocial* - RAPS),^16^ which includes primary health care and rehabilitation services, features significant care gaps, both geographically and in terms of clinical conditions/diagnosis.

In a study on the effective implementation of RAPS in health regions,^17^ it was identified that, of the 438 health regions in Brazil, more than a third are characterized by low socioeconomic development and low supply of services. The North and Northeast regions had the highest percentages of health regions with a low supply of services,^17^ contributing to explain the lower prevalence of reports of depression diagnosis. Concerning the capacity of primary care teams, Gerbaldo et al.^18^ showed higher turnover of professionals and lower proportions of professionals who reported being prepared for mental health demands in the North and Northeast regions, in contrast to the Southeast and South regions. In addition, the North, Northeast and Midwest regions showed worse results in all indicators of mental health care provision, in contrast with the Southeast and South regions, revealing significant inequalities in the organization and structuring of health services in these regions.

Regarding the sociodemographic profile, the higher prevalence of self-reported depression among females is consistent with national and international literature,^19^ ^,^ ^20^ which indicate that women are twice as likely to develop depression in the course of their lives than men. These differences are associated with biological (sex) and sociocultural (gender) aspects related to the identification of symptoms and seeking help for psychiatric disorders in general and for depressive symptoms, in particular.^21^ ^,^ ^22^

In relation to level of education, higher prevalence among people at the extremes of the education ranges is consistent with the literature, which points to a higher incidence of mental disorders both among individuals with low socioeconomic and education levels^23^ ^,^ ^24^ and those with higher levels of education and greater access to information on health and to private health cares and health insurance.^25^ ^,^ ^26^

Literature on the relationship between race/skin color and mental health in Brazil is scarce, but does not corroborate the present study, which identified a higher prevalence in people who self-declare as White. In a recent systematic review article, Smolen and Araújo^27^ identified a trend towards a higher prevalence of mental disorders in Non-White individuals. This difference between results in studies on race/color/ethnicity and mental health has been the focus of investigation, especially in the United States population, which identified that psychological distress is more frequent in Black individuals, despite not meeting the diagnostic criteria for major depression.^28^

In Brazil, the self-report of color/race/ethnicity is a complex variable, which includes genetic and phenotypic as well as psychological, socioeconomic and cultural aspects and, therefore, is affected by processes of change in racial identification related to the improvement of living conditions of the Black population, also involving regional and generational differences.^27^ Therefore, taking into account that the measure of depression in the present study was self-reported, it is possible that the Black population, which has a higher proportion of individuals with lower education, income and access to health services,^25^ has contributed to a higher prevalence of depression among individuals who report being White.

Concerning the use of health services, the increase in the prevalence of individuals with self-reported depression medical diagnosis who received medical care in the 12 months prior to the interview, especially among young adults (18 to 29 years old) and urban residents, is consistent with data of a Brazilian study in a birth cohort of adolescents and young adults, which identified higher prevalence of current major depressive episode in young adults,^23^ and with studies on university students,^29^ reinforcing the importance of further studies on mental health focusing on this age group.

Considering the scarcity of population-based and nationwide surveys to assess the prevalence of depression, the present study provides relevant information on the distribution of self-reported depression in the Brazilian population and related sociodemographic factors, as well as on the availability and access to diagnosis and treatment in RAPS. Furthermore, the comparison with the 2013 PNS allowed for the analysis of the prevalence of self-reported depression over the years.

This study, however, has limitations that must be considered. It is likely that the restriction of sample selection among domiciled individuals underestimates the prevalence of depression, since populations in situations of extreme vulnerability (homeless, internally displaced, deprived of liberty, hospitalized, etc.) are at greater risk of being affected by mental disorders and psychological distress and were not included in the sample. Moreover, the prevalence of self-reported depression is a limited indicator for estimating the prevalence of depression in the population, which should ideally be measured by means of standardized and validated diagnostic instruments or clinical evaluations. In this regard, the results presented herein should not be understood as the prevalence of depressive disorders in the population, but should be interpreted in the light of inequities in access to mental health services in the country, which are instrumental for the access to the medical diagnosis of depression.

Aspects related to the influence of psychosocial dimensions (gender, race/color/ethnicity, life cycle, among others) on comprehending the health-disease process, the self-perception of depressive symptoms and on the training of health professionals to identify psychiatric symptoms were not explored in this study and may be related to the pursuit of medical care and to the available treatments. In this regard, it is suggested that these issues be investigated in research on the use of health services.

The use of validated screening instruments for the identification of depressive symptoms, anxiety disorders and disorders resulting from alcohol abuse in recurrent population surveys can better estimate the specific prevalence of such disorders, reducing the uncertainty resulting from the variability surrounding the self-reported previous medical diagnosis, thus monitoring and increasing surveillance of these disorders, which are highly prevalent in the general population.

In essence, depression is characterized as a highly prevalent disorder in the Brazilian population. The increase in the prevalence of self-reported depression may result from the increase in health care coverage in the period, data which is reinforced by the growth in the proportion of individuals diagnosed with depression who received medical care in the 12 months preceding the interview. Demographic differences in the prevalence, on the other hand, draw attention to disparities in access to mental health services for diagnosis. The high proportion of individuals resorting to private doctors for treatment indicates a fragility in the equitable access to mental health services by the Brazilian population.

Lastly, the execution of nationwide epidemiological studies employing standardized diagnostic instruments, and which also permit the investigation of risky behaviors and exposure to adverse events, could contribute to the definition of a better panorama of the Brazilian population’s mental health, laying a solid foundation for mental health policies in Brazil.
